# What is the best treatment strategy for primary spontaneous pneumothorax? A retrospective study

**DOI:** 10.1016/j.amsu.2019.07.034

**Published:** 2019-08-03

**Authors:** Yasuhiro Chikaishi, Masatoshi Kanayama, Akihiro Taira, Yusuke Nabe, Shinji Shinohara, Taiji Kuwata, Ayako Hirai, Naoko Imanishi, Yoshinobu Ichiki, Fumihiro Tanaka

**Affiliations:** a Second Department of Surgery, School of Medicine, University of Occupational and Environmental Health, 1-1 Iseigaoka, Yahatanishi-ku, Kitakyushu, 807-8555, Japan; b Department of Chest Surgery, Shimonoseki City Hospital, 1-13-1 Kouyouchou, Shimonoseki, 750-8520, Japan

**Keywords:** Pneumothorax, Recurrence, Surgery, PSP, primary spontaneous pneumothorax, CT, computed tomography

## Abstract

**Background:**

Several treatment strategies are available for primary spontaneous pneumothorax (PSP). Surgical procedures are also performed in patients with PSP without an absolute indication for surgery. This study was performed to investigate the best treatment strategy for PSP by comparison of the recurrence rate.

**Materials and methods:**

From January 2006 to December 2013, 149 patients with PSP aged ≤50 years were treated in our institution. We reviewed the recurrence rate of PSP for each treatment strategy and evaluated the association between the recurrence rate of PSP with the clinicopathological characteristics. We also compared the surgery and non-surgery groups.

**Results:**

A significant difference in the PSP recurrence rate was found between the surgery and non-surgery groups (22% vs. 52%, respectively; p < 0.001), patients aged ≥22 and < 22 years (16% vs. 44%, respectively; p < 0.001), and smokers and nonsmokers (13% vs. 43%, respectively; p < 0.001). There were also significant differences in the multivariate analysis (p < 0.001, p = 0.050, and p = 0.001, respectively). In the surgery group, the PSP recurrence rate was significantly different between patients aged ≥22 and < 22 years (7% vs. 38%, respectively; p < 0.001) and smokers and nonsmokers (5% vs. 33%, respectively; p = 0.002). No significant differences were found in the non-surgery group.

**Conclusions:**

In the surgical treatment of PSP, it is desirable that smokers stop using tobacco and that patients are ≥22 years old. Moreover, when surgery is being considered, the best timing seems to be when air leakage is present because the air leakage sites can be resected.

## Highlights

• Younger patients had a higher recurrence rate than older patients.
• Smokers had a lower recurrence rate than nonsmokers.
• When we select the surgical treatment of PSP, it is desirable that smokers stop using tobacco and that patients are older.

## 1 Introduction

Primary spontaneous pneumothorax (PSP) occurs at frequency of 7.4–18.0 per 100,000 men and 1.2 to 6.0 per 100,000 women [1,2]. In general, the surgical indications for PSP are as follows [3]: a second episode of PSP, persistent air leakage for longer than 3–5 days, bilateral pneumothorax, significant hemothorax, and professions at risk (e.g., aircraft personnel and divers). Patients with PSP undergo operations if they have a surgical indication, such as persistent air leakage, bilateral pneumothorax, or significant hemothorax. The consensus is that surgical treatment should be avoided if conservative treatment is possible [2,4,5]. However, some patients with PSP undergo operations without an absolute indication for surgery, such as those who desire to undergo surgery to reduce the risk of recurrence. Whether surgery should be performed for patients with PSP without an absolute indication for surgery remains controversial. To the best of our knowledge, no studies have compared surgical treatment with conservative treatment for patients with PSP. We hope to establish the best treatment procedure for PSP by comparison of surgical and conservative treatment. The purpose of this study was to investigate the best treatment strategy for patients with PSP by comparison of the recurrence rate associated with each strategy.

## 2 Materials and methods

We retrospectively reviewed patients with PSP aged <50 years who underwent treatment in our institution from January 2006 to December 2013. Patients with simultaneous bilateral pneumothorax, lung disease, and traumatic and iatrogenic etiologies were excluded.

The therapeutic strategy for PSP in our institution is to follow-up patients with mild collapse and perform chest drainage for those with moderate or severe collapse. In general, we do not perform surgery and pleurodesis for patients with PSP without an absolute indication for surgery. Collapse was classified into three patterns according to Kircher's method [6]: mild collapse, <20%; moderate collapse, 20%–50%; and severe collapse, >50%. Operations were performed in patients with surgical indications for PSP, as mentioned above. However, even in patients with no air leakage and full lung expansion, we sometimes performed an operation if the patients desired it. For patients who underwent operations, we performed chest computed tomography (CT) if at all possible, and all operations involved the video-assisted thoracoscopic surgery approach.

We studied the recurrence rate of PSP with respect to surgery, age, sex, left or right affected side, smoking, collapse level, and previous illness associated with contralateral pneumothorax. The presence or absence of recurrence was confirmed by the clinical records of April 2015. We also compared these factors between patients in the surgery and non-surgery groups. In the surgery group, we also examined the reason for the surgery and the presence and location of air leakage sites in the lung by referring to the surgical record. In the non-surgery group, we also examined the therapeutic strategy and presence of bullae on chest CT.

Statistical analysis was performed with the StatView 5.0J software package (SAS Institute, Cary, NC, USA). Categorical variables were analyzed using the chi-square test. Multivariate analysis was performed using a logistic regression model. A p value of <0.05 was considered statistically significant.

## 3 Results

In total, 149 patients with PSP who had been treated in our institution were retrospectively studied. The patients’ characteristics are listed in Table 1. Their mean age was 22 years (range, 14–50 years). There were 136 male patients (91.3%) and 13 female patients (8.7%). A total of 101 patients underwent surgery, and the postsurgical drainage period ranged from 1 to 13 days (median, 2 days). The involved chest side was the left in 85 patients (57%) and right in 64 (43%). Fifty-five (36.9%) patients were smokers, 90 (60.4%) were nonsmokers, and 4 (2.7%) had an unknown smoking history. Thirty-one (20.8%) patients had a history of contralateral PSP.

**Table 1:** Characteristics of all patients with PSP.

Characteristic	Total	Rec^a^	%	Non-Rec^b^	%	*p*-value
All cases	149	46	31	103	69	
Gender						
Male	136	42	31	94	69	0.993
Femail	13	4	31	9	69	
Age (year)						
<22	75	33	44	42	56	<0.001
≧22	74	13	18	61	82	
Surgery						
Yes	101	21	21	80	79	<0.001
no	48	25	52	23	48	
Lesion site						
Left	85	27	32	58	68	0.786
Right	64	19	30	45	70	
Smoking						
Yes	55	7	13	48	87	<0.001^a^
No	90	39	43	51	57	
Unknown	4	0	0	4	100	
Collapse level						
Mild	14	6	43	8	57	0.518
Middle	90	28	31	62	69	
Severe	45	12	27	33	73	
CSP^c^						
Yes	31	11	35	20	65	0.532
No	118	35	30	83	70	

PSP = primary spontaneous pneumothorax, Rec = recurrence, CSP = contralateral spontaneous pneumothorax.

Percentages calculated are row-wise.

Categorical variables were analyzed using the chi-square test.

a The four unknown patients in the smoking group were omitted from calculation of the p value.

Table 1 also shows that there was a significant difference in the PSP recurrence rate between the surgery and non-surgery groups (22% vs. 52%, respectively; p < 0.001), patients aged ≥22 and < 22 years (16% vs. 44%, respectively; p < 0.001), and smokers and nonsmokers (13% vs. 43%, respectively; p < 0.001). These factors were also significantly different in the multivariate analysis (p < 0.001, p = 0.050, and p = 0.001, respectively) (Table 2). There was no significant difference in sex, right and left sides, collapse level, or presence of contralateral pneumothorax. Table 3 shows the characteristics of the surgery group. A significant difference in the pneumothorax recurrence rate was found between patients aged ≥22 and < 22 years (7% vs. 38%, respectively; p < 0.001) and between smokers and nonsmokers (5% vs. 33%, respectively; p = 0.002). Moreover, although there was no significant difference, the tendency for recurrence was lower in the patients with than without air leakage sites in the lung during surgery. Age was shown to be significantly different in the multivariate analysis (p = 0.022) (Table 4). There was no significant difference in the reason for surgery, collapse level, or presence of contralateral pneumothorax. Table 5 shows the characteristics of the non-surgery group. There was no significant difference in the therapeutic strategy, presence of bullae on CT, age, smoking, collapse level, or presence of contralateral pneumothorax. However, there tended to be a difference between smokers and nonsmokers (33% vs. 61%, respectively; p = 0.080).

**Table 2:** Multivariate analysis of all patients with PSP.

Variables	Characteristics		multivariate analysis			
	Unfavorable	Favorable	CE.	S.E.	OR	*p* value
Age (y)	≧22	<22	−0.844	0.431	0.430	0.05
Surgery	no	yes	1.391	0.408	4.017	<0.001
Smoking	Negative	Positive	−1.283	0.506	0.277	0.001

Multivariate analyses of factors associated with recurrence were performed using a logistic regression model.

CE = coefficient, S.E. = standard error, OR = odds ratio.

**Table 3:** Characteristics of patients with PSP in the surgery group.

Characteristic	Total	Rec^a^	%	Non-Rec^b^	%	*p*-value
All cases	101	21	21	80	79	
Age (year)						
<22	45	17	38	28	62	<0.001
≧22	56	4	7	52	93	
reason						
leakage	60	10	17	50	83	0.426
hemopneumothrax	6	2	33	4	67	
others	35	9	26	26	74	
leak point						
clear	35	4	11	31	89	0.091
unknown	66	17	26	49	74	
Smoking						
Yes	40	2	5	38	95	0.002^a^
No	57	19	33	38	67	
Unknown	4	0	0	4	100	
Collapse level						
Mild	6	1	17	5	83	0.945
Middle	59	12	20	47	80	
Severe	36	8	22	28	78	
CSP^c^						
Yes	27	8	30	19	70	0.186
No	74	13	18	61	82	

PSP = primary spontaneous pneumothorax, Rec = recurrence, CSP = contralateral spontaneous pneumothorax.

Percentages calculated are row-wise.

Categorical variables were analyzed using the chi-square test.

a The four unknown patients in the smoking group were omitted from calculation of the p value.

**Table 4:** Multivariate analysis of the surgery group.

Variables	Characteristics		multivariate analysis			
	Unfavorable	Favorable	CE.	S.E.	OR	*p* value
Age (y)	≧22	<22	−1.479	0.646	0.905	0.022
leak point	no	yes	−0.864	0.648	0.422	0.183
Smoking	Negative	Positive	−1.579	0.829	0.206	0.057

Multivariate analyses of factors associated with recurrence were performed using a logistic regression model.

CE = coefficient, S.E. = standard error, OR = odds ratio.

**Table 5:** Characteristics of patients with PSP in the non-surgery group.

Characteristic	Total	Rec^a^	%	Non-Rec^b^	%	*p*-value
All cases	48	25	52	23	48	
Age (year)						
<22	29	16	55	13	45	0.597
≧22	19	9	47	10	53	
procedure						
rest	6	4	67	2	33	0.445
dranage	42	21	50	21	50	
bullae in CT						
yes	38	19	50	19	50	0.383
no	9	6	67	3	33	
n.d.	1	0	0	1	100	
Smoking						
Yes	15	5	33	10	67	0.08
No	33	20	61	13	39	
Collapse level						
Mild	8	5	63	3	38	0.755
Middle	31	16	52	15	48	
Severe	9	4	44	5	56	
CSP^c^						
Yes	4	3	75	1	25	0.338
No	44	22	50	22	50	

PSP = primary spontaneous pneumothorax, Rec = recurrence, CSP = contralateral spontaneous pneumothorax.

Percentages calculated are row-wise.

Categorical variables were analyzed using the chi-square test.

## 4 Discussion

The results of the present study are notable for two reasons. First, this study examined patients with PSP, including both patients who did and did not undergo surgery. Some recent studies [7,8], including our previous study [9], were performed to evaluate the postoperative recurrence rate; conservative cases were not included in these studies. Few studies have surveyed patients with PSP in both surgery and non-surgery groups [10]. We believe that surveillance of both surgery and non-surgery groups will help to establish a PSP treatment strategy. Second, to avoid bias, we excluded patients with complicated diseases such as simultaneous bilateral pneumothorax, lung disease, and traumatic and iatrogenic etiologies. This allowed us to evaluate the pure recurrence rate of PSP.

The present study demonstrated five major findings. First, the recurrence rate was lower after surgery than after conservative therapy. In general, the first-choice treatment strategy for PSP is conservative therapy [2,5]. However, surgery reduces the recurrence rate of PSP at the same time and cost performance as conservative therapy [10]. Other studies have evaluated the surgical strategy for PSP [9]. In the present study, the recurrence rate was lower after surgery than after conservative therapy.

Second, younger patients had a higher recurrence rate than older patients. Few reports have mentioned that younger patients have a tendency to develop PSP recurrence [11]. The higher recurrence rate might be because young patients have more immature lung tissue. A lower recurrence rate in younger patients is controversial [9,11].

Third, smokers had a lower recurrence rate than nonsmokers. In our previous institutional report, which addressed surgical patients with PSP, we described a similar result [9]. This finding might seem contrary to logic because cigarette smoking is generally associated with the occurrence of pneumothorax [12,13]. However, we obtained a different result in the present study. Pneumothorax in smokers occurs through reversible changes such as bronchiolitis [13]; in contrast, pneumothorax in nonsmokers occurs through irreversible changes such as bullae formation. One hypothesis states that the reversible changes in smokers, such as bronchiolitis, improve by stopping tobacco use, which in turn reduces the recurrence rate [14].

Fourth, among patients who underwent conservative therapy, there was no significant difference in the recurrence rate according to age or the presence of bullae. Because the recurrence rate associated with conservative therapy itself was higher than that associated with surgery, our evaluation may not accurately reflect the real-world situation. In general, however, the presence of bullae contributes to recurrence [15]. In the present study, conservative therapy was associated with a high recurrence rate with or without bullae.

Finally, the tendency for recurrence was lower in the patients with than without air leakage sites in the lung during surgery. Perhaps the responsible lesion was not resected in patients without air leakage. This result indicates that it is important to find the responsible lesion during surgery if at all possible.

This study has several limitations. It was retrospective in design and performed at a single institution. There was bias in the follow-up time because patients with pneumothorax do not visit a doctor if they have been successfully treated, unlike patients with malignant disease such as carcinoma. Therefore, we did not perform follow-up if patients with recurrence visited another doctor. Because this was a retrospective study, the surgical indications slightly differed depending on the doctor.

## 5 Conclusions

In conclusion, in the surgical treatment of PSP, it is desirable that smokers stop using tobacco and that patients are ≥22 years old. Moreover, when surgery is being considered, the best timing seems to be when air leakage is present because the air leakage sites can be resected.

## Ethics approval

Ethics approval for this study was obtained from the ethics review committee for human studies of the University of Occupational and Environmental Health. This manuscript has been reviewed and approved.

By all the co-authors and has not been submitted to any other journals for consideration for publication.

## Sources of funding

The authors declare no financial support.

## Author contribution

Yasuhiro Chikaishi; Writing the paper, Study design. Masatoshi Kanayama; Others, Akihiro Taira; Other, Yusuke Nabe; Other, Shinji Shinohara; Other, Taiji Kuwata; Other, Ayako Hirai; Other, Naoko Imanishi; Other, Yoshinobu Ichiki; Other, Fumihiro Tanaka; Study design, and all authors read and approved the final manuscript.

## Conflicts of interest

None.

## Research registration number

Research Registration Unique Identifying Number is researchregistry4774.

## Availability of data and materials

The datasets used are available from the corresponding author on reasonable request.

### Provenance and peer review

Not commissioned, externally reviewed.

## Trial registry number

Non applicable.

## Guarantor

Fumihiro Tanaka, M.D., Ph.D.
